# Understanding the Agility of Running Birds: Sensorimotor and Mechanical Factors in Avian Bipedal Locomotion

**DOI:** 10.1093/icb/icy058

**Published:** 2018-06-12

**Authors:** Monica A Daley

**Affiliations:** Structure and Motion Lab, Royal Veterinary College, Hawkshead Lane, Hertfordshire, AL9 7TA, UK

## Abstract

Birds are a diverse and agile lineage of vertebrates that all use bipedal locomotion for at least part of their life. Thus birds provide a valuable opportunity to investigate how biomechanics and sensorimotor control are integrated for agile bipedal locomotion. This review summarizes recent work using terrain perturbations to reveal neuromechanical control strategies used by ground birds to achieve robust, stable, and agile running. Early experiments in running guinea fowl aimed to reveal the immediate intrinsic mechanical response to an unexpected drop (“pothole”) in terrain. When navigating the pothole, guinea fowl experience large changes in leg posture in the perturbed step, which correlates strongly with leg loading and perturbation recovery. Analysis of simple theoretical models of running has further confirmed the crucial role of swing-leg trajectory control for regulating foot contact timing and leg loading in uneven terrain. Coupling between body and leg dynamics results in an inherent trade-off in swing leg retraction rate for fall avoidance versus injury avoidance. Fast leg retraction minimizes injury risk, but slow leg retraction minimizes fall risk. Subsequent experiments have investigated how birds optimize their control strategies depending on the type of perturbation (pothole, step, obstacle), visibility of terrain, and with ample practice negotiating terrain features. Birds use several control strategies consistently across terrain contexts: (1) independent control of leg angular cycling and leg length actuation, which facilitates dynamic stability through simple control mechanisms, (2) feedforward regulation of leg cycling rate, which tunes foot-contact timing to maintain consistent leg loading in uneven terrain (minimizing fall and injury risks), (3) load-dependent muscle actuation, which rapidly adjusts stance push-off and stabilizes body mechanical energy, and (4) multi-step recovery strategies that allow body dynamics to transiently vary while tightly regulating leg loading to minimize risks of fall and injury. In future work, it will be interesting to investigate the learning and adaptation processes that allow animals to adjust neuromechanical control mechanisms over short and long timescales.

## Birds as an animal model for agile bipedal locomotion

Birds are diverse and agile vertebrates capable of many combinations of aerial, terrestrial, and aquatic locomotion. Living birds vary in size from hummingbirds to ostriches, and exhibit diversity in the length and mass proportions of the wings and legs, reflecting adaptation for different locomotor ecologies (Gatesy and Middleton 1997; Zeffer et al. 2003; Heers and Dial 2015). While wings and flight are a defining locomotor innovation of birds, many living bird species are impressive bipedal terrestrial athletes, and all birds use bipedal movement for at least some part of their lives (Abourachid and Höfling 2012; Heers and Dial 2015). Birds inherited bipedalism and many hindlimb morphological features from theropod dinosaurs, an ancient lineage that first appeared around 230 million years ago (Gatesy and Middleton 1997). This diversity and bipedal legacy makes birds a valuable study system for investigating how morphology, biomechanics, and sensorimotor control are integrated for agile bipedal locomotion.

## What are the challenges in achieving agile bipedal locomotion?

Legged locomotion is complex and dynamic, involving abrupt foot-contact transitions and uncertainty due to variable terrain and sensorimotor errors. Animals must precisely control limb dynamics to move effectively over varied and uncertain terrain while avoiding falls, collisions, and injury (Daley 2016). It remains poorly understood how the sensory, neural, and mechanical components of control are integrated to achieve robust, stable, and agile locomotion. Here, robustness refers to how large a disturbance an animal can tolerate while still meeting the functional demands of the task, such as forward movement at an acceptable speed (Daley 2016). Disturbances can arise externally from the environment, internally from sensorimotor noise (such as errors in motor commands), or from inaccurate sensory information (such as lack of visibility or conflict between sensory modalities). Stability quantifies how rapidly the system attenuates perturbations from steady-state locomotion, and agility refers to the ability to rapidly adjust locomotor dynamics to meet changing task demands (such as a rapid extension of the leg to leap over an obstacle) (Daley 2016; Duperret et al. 2016). Locomotion must be robust, stable, and agile for effective locomotion in natural conditions.

Avoiding slip, fall, and injury requires precise regulation of foot-contact timing and leg-substrate interaction forces (Alexander 2002; Clark and Higham 2011; Birn-Jeffery and Daley 2012; Daley 2016). Yet, inherent uncertainty due to terrain variability, sensorimotor noise, and sensing errors mean that the system dynamics cannot be perfectly sensed or predicted. Considering these challenges, the agility and robust stability of terrestrial animals is truly remarkable. Bipedal animals face the additional challenge that they have fewer legs to support the body compared to quadrupeds and other many-legged animals. Quadrupeds can redistribute loads among the legs in response to perturbations—a strategy not available to a rapidly running biped. This likely makes the challenges for dynamic balance control, especially acute for bipedal animals.

One inherent challenge of animal systems is sensorimotor delay that limits feedback response times (More et al. 2010; 2013). Sensorimotor delays include delays from sensing, nerve transmission, synapses, muscle electromechanical coupling, and muscle force development (More et al. 2013). Delays necessitate the use of predictive feedforward control, because motor commands must be issued in advance of the required mechanical demands. Reactive feedback control is also crucial to modulate and update motor commands to correct for deviations between predicted and actual dynamics. Thus, sensorimotor delay necessitates that animals effectively integrate both predictive (feedforward) and reactive (feedback mediated) sensorimotor control mechanisms for effective locomotion (Rossignol et al. 2006; Ijspeert 2018).

**Fig. 1.**
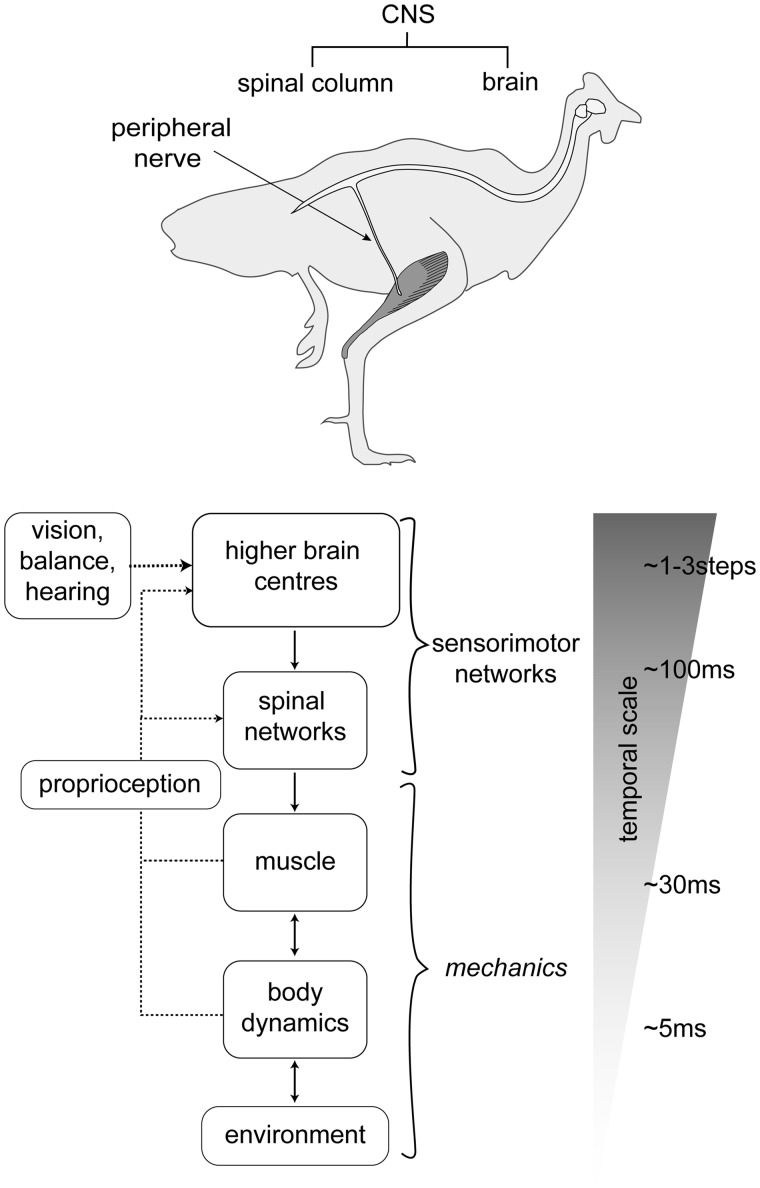
Schematic illustration of the hierarchical organization of vertebrate neuromechanical control. Transmission delays lead to a temporal scaling of sensorimotor processes that relate to the anatomical distances between sensors, neural networks and effectors. Consequently, central, peripheral and mechanical mechanisms must be integrated over short and long timescales. The fastest responses occur in the periphery, through intrinsic mechanics, intermediate responses occur through short-latency spinal reflexes, and slower responses involve processing and planning in higher brain centers.

Nerve transmission delays increase with the anatomical distances of neural pathways. This physical constraint creates a direct link between neuroanatomy and temporal scaling of control processes (Fig. 1). Considering this, delay has probably been a selective factor in the evolution of a hierarchical organization of the nervous system. The fastest possible reactions occur locally, through intrinsic-mechanical responses to altered limb-substrate interactions (Brown and Loeb 2000; Daley and Biewener 2006; Daley et al. 2006; 2009). The shortest sensorimotor loops and fastest neural responses occur through monosynaptic spinal reflexes, and the longest delays are associated with processing and predictive planning in higher brain centers (Fig. 1) (Rossignol et al. 2006; Grillner et al. 2008; McCrea and Rybak 2008; McLean and Dougherty 2015; Kiehn 2016). This suggests the natural emergence of temporal scaling of sensorimotor control that relates to neuroanatomical organization. While the components of vertebrate sensorimotor systems are increasingly well understood, it remains unclear how these mechanisms are integrated over varying timescales to achieve robust, stable, and agile locomotion in natural terrain contexts.

The phrase “passive-dynamics” has often been used to refer to the intrinsic mechanical response of the locomotor system. However, this phrase can be somewhat misleading, because the intrinsic mechanical response is actively tuned by the selection of a specific muscle activation pattern from the possible solutions that could meet the mechanical requirements of the task (Brown and Loeb 2000). Each muscle activation solution will confer a unique set of characteristics in terms of muscle–tendon dynamics, impedance response, stability, robustness and sensitivity to perturbations, directional tuning, and energy cost (Brown and Loeb 2000; Inouye and Valero-Cuevas 2016; Valero-Cuevas 2016). Thus, intrinsic mechanical responses are under some active control, because feedforward muscle activation patterns can be tuned through learning and experience to enable robust, stable, and agile performance. However, the processes and timescales of such tuning between intrinsic mechanics and muscle activation patterns remain unclear.

## Terrain perturbation approaches help reveal neuromechanical control strategies

Terrain perturbations are ubiquitous in nature and disrupt the predictability and timing of foot–substrate interactions, requiring transient locomotor responses to recover from disturbances. Understanding transient locomotor dynamics is important for revealing natural locomotor behaviors, and for understanding the specific mechanical demands and constraints that have shaped animal locomotor control.

Birds are particularly useful for such studies of transient locomotor dynamics, because it is possible to simultaneously measure *in vivo* muscle force–length dynamics, body dynamics, leg–substrate interaction forces, and joint mechanics during locomotion (Daley and Biewener 2006; Daley et al. 2007, 2009). This facilitates integrated understanding of neuromechanical function. Considering the complex nature of neuromechanical control, it is useful to start by investigating the response to very simple terrain perturbation features, to minimize the number of confounding factors in the response.

**Fig. 2.**
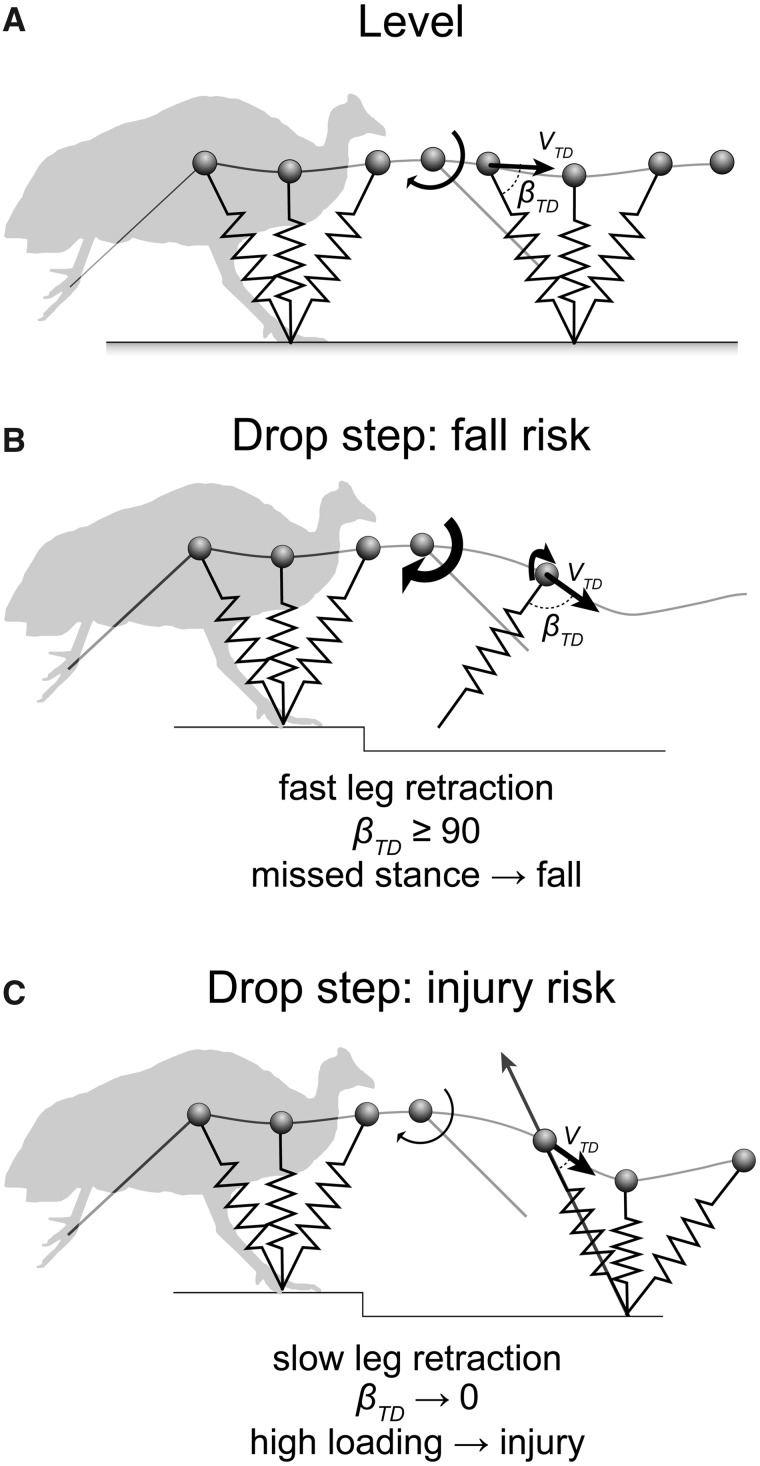
A trade-off in control of leg retraction rate for terrain robustness versus injury avoidance, illustrated by two boundary conditions. (A) Running dynamics modeled as a SLIP with the swing leg retracted toward the ground just before stance. Leg retraction rate influences the mechanical response in uneven terrain: (B) Fast leg retraction results in steeper leg contact angles and minimizes fluctuations in leg loading, but if leg loading angle (β_TD_) reaches 90-degrees, the leg will miss stance, risking a fall. Maximum terrain drop before a fall decreases with increasing rate of leg retraction. (C) Slow leg retraction ensures leg contact, minimizing fall risk, but incurs higher fluctuations in leg loading. Evidence suggests that birds optimize their leg retraction rate to minimize fluctuations in leg-loading (Blum et al. 2014), using intermediate leg retraction rates that ensure contact while avoiding overload injury.

Initial terrain perturbation experiments in running guinea fowl aimed to reveal the immediate intrinsic mechanical response to an unexpected perturbation, in the time before a sensorimotor feedback response is possible (Daley and Biewener 2006; Daley et al. 2007, 2009). This work was inspired by earlier work in rapidly running cockroaches recovering from an impulsive perturbation (Jindrich and Full 2002) and studies of humans recovering from sudden changes in terrain stiffness (Ferris et al. 1999; Moritz and Farley 2004). In the guinea fowl experiments, birds encountered a simple camouflaged pothole step, 8 cm deep (40% of leg length), covered by opaque tissue paper stretched across the gap (Daley et al. 2006). When navigating the unexpected drop, guinea fowl showed large changes in leg posture in the perturbed stance, which correlated strongly with leg loading and perturbation recovery. These findings highlighted the role of leg angular trajectory control for regulating foot contact timing and leg loading (Daley and Biewener 2006), which has also been found to be important in humans (Seyfarth et al. 2003; Daley and Biewener 2006). The dynamics following a drop in terrain can be explained by the physics of a spring-loaded-inverted pendulum (SLIP) model with very simple swing leg control, in which the leg follows a sinusoidal, clock-like angular trajectory, retracting backwards toward the ground just before the swing-stance transition (Fig. 2; Seyfarth et al. 2003; Daley and Biewener 2006; Blum et al. 2014). In this model, contact angle depends on the duration of the ballistic flight time (Daley and Biewener 2006; Daley and Usherwood 2010; Blum et al. 2014). Although this is an extremely simplistic model of running, it is sufficient to generate robustly stable gait dynamics (Seyfarth et al. 2003; Blum et al. 2014).

Such model-based analyses of the coupling of body dynamics and leg angular cycling during running over terrain perturbations have revealed an inherent trade-off in leg control between terrain robustness and injury avoidance (Fig. 2; Daley and Usherwood 2010; Blum et al. 2014). Drops in terrain result in a delay of ground contact, longer fall time, and greater downward vertical velocity at contact. However, the specific dynamics of the response depends on how fast the leg is retracted during the falling phase, just before the swing-stance transition (Daley and Usherwood 2010). Fast leg retraction results in a large change in leg angle in response to a given change in terrain height, and earlier ground contact. This results in smaller fluctuations in vertical velocity and leg loading in response to terrain perturbations (Daley and Usherwood 2010; Blum et al. 2014). However, fast leg retraction also decreases the maximum terrain drop the animal can safely negotiate, a measure of robustness, and increases the risk that the leg will miss contact entirely, leading to a fall (Fig. 2B;Daley and Usherwood 2010; Blum et al. 2014). In contrast, slow leg retraction results in small changes in leg angle for a given terrain drop, ensuring foot contact even for large terrain perturbations, reducing risk of fall; however, this leads to larger increases in vertical velocity and leg loading in the stance following the drop, increasing risk of overload injury (Fig. 2C).

Subsequent experiments have investigated how leg control strategies vary depending on the type of perturbation (pothole, step, obstacle), and with ample practice negotiating visible terrain features. In comparing locomotor control strategies between hidden and visible potholes, guinea fowl slow down in anticipation of visible potholes when they encounter them for the first time, and actually stumble more when negotiating the visible drop (Daley and Biewener 2006). Although the high-speed intrinsic-mechanical response to an unexpected drop is robustly stable, birds may not always choose this strategy when they first encounter novel, visible terrain features, perhaps to minimize risk of injury.

Blum and colleagues (2014) explored how animals manage the trade-off between terrain robustness and injury avoidance in leg angular control when given ample practice negotiating a visible drop in terrain. Under these conditions, guinea fowl converge upon a strategy similar to the hidden pothole strategy—they maintain high speeds and allow intrinsic leg mechanics to mediate the perturbation response (Blum et al. 2014). The authors compared the experimental measures to SLIP-model predictions with swing leg angular control optimized for disturbance rejection (robustness) versus load regulation (injury avoidance). The guinea fowl used a strategy that allowed body dynamics to transiently vary, with swing leg control optimized to maintain consistent leg loading in uneven terrain, which avoids both fall and injury conditions. Model analysis revealed that leg control optimized for disturbance rejection, to maintain steady body dynamics, demanded dramatic increases in leg loading, suggesting increased injury risk. This study also revealed that birds showed very little stride-to-stride variance in leg angular cycling rate in uneven terrain. In contrast, leg length actuation rapidly changed in response to altered leg posture and loading, resulting in rapid adjustment of stance push-off to stabilize body mechanical energy in the 1–2 steps following the perturbation (Blum et al. 2014). These studies have revealed optimization of leg angular cycling rate as an effective control strategy for locomotion in uneven terrain, allowing maintenance of consistent leg loading and high running speeds (Seyfarth et al. 2003; Daley and Biewener 2006; Daley and Usherwood 2010; Blum et al. 2011, 2014).

**Fig. 3.**
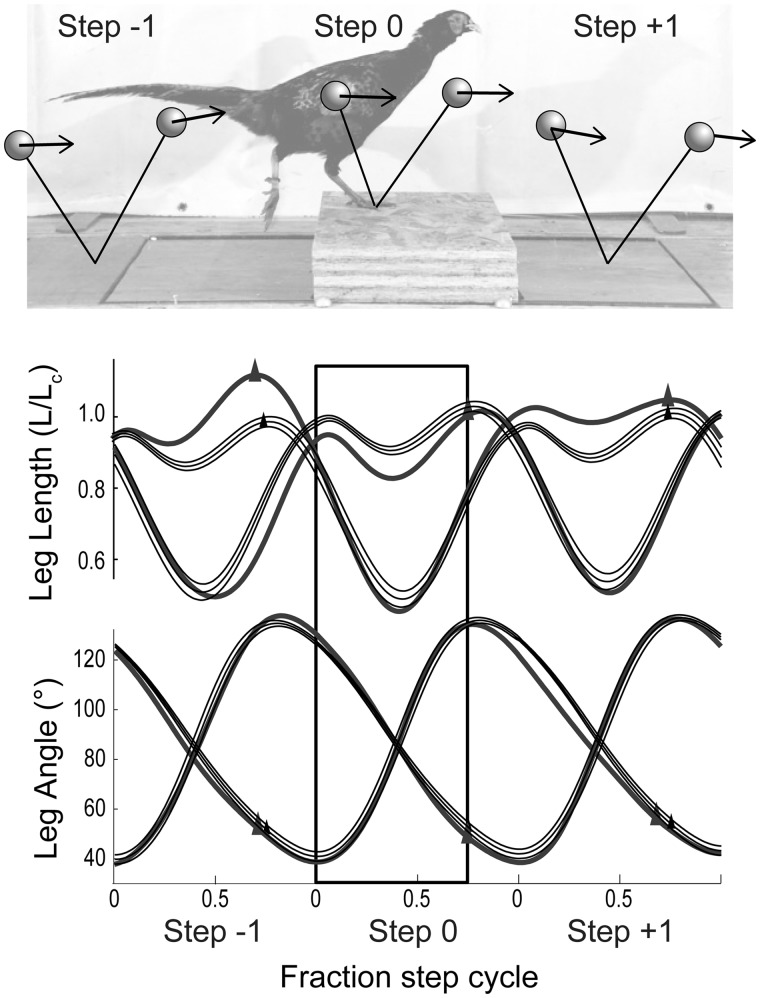
Leg length and leg angular trajectories of pheasants negotiating visible obstacles, illustrating a typical three-step strategy. At top, schematic illustration of the landing and take-off conditions of the bird during the step preceding (Step −1), the step on the obstacle (Step 0), and the obstacle dismount (Step +1). Below, leg trajectory (length and angle) during running on level terrain (thin black lines, mean and 95% confidence intervals) and over an obstacle height of 30% leg length (thicker gray lines). Upward triangles indicate foot take-off at the end of stance. Leg length exhibits high stride-to-stride variance in uneven terrain, whereas leg angular trajectory follows a relatively consistent sinusoidal trajectory, with only subtle changes in rate in anticipation of terrain height changes. Data from Birn-Jeffery and Daley (2012).

In another series of experiments, Birn-Jeffery and colleagues investigated control strategies used by ground birds when negotiating visible obstacles, to investigate potential trade-offs in stance leg function (Birn-Jeffery and Daley 2012; Birn-Jeffery et al. 2014). Similar to the studies on terrain drops, the birds exhibited independent control of leg angular cycling and leg length trajectory, with higher stride-to-stride variance in leg length in uneven terrain (Fig. 3) When running over a visible obstacle, birds use a three-step negotiation strategy, with clear evidence of feedforward, predictive adjustments in the step preceding the obstacle (Fig. 3). Model-based analyses suggest that the strategy used by birds is most consistent with models optimized to regulate leg loading in uneven terrain, not to maintain steady body dynamics (Birn-Jeffery et al. 2014).

Regulation of leg cycling rate can be viewed as a combined feedforward plus ‘preflexive’ control strategy that minimizes the need for reactive adjustments by exploiting the intrinsic mechanical coupling between leg contact angle and leg loading. Experimental evidence from both humans and birds running over a range of terrain perturbations are consistent with leg angular trajectory as a key target of neural control (Seyfarth et al. 2003; Blum et al. 2011; Müller et al. 2016). Humans and birds allow body dynamics to transiently vary, but exhibit tight coupling between leg contact angle and leg loading across many different terrain contexts (Grimmer et al. 2008; Birn-Jeffery and Daley 2012; Birn-Jeffery et al. 2014; Blum et al. 2014; Müller et al. 2016). Empirical evidence from birds running over a range of terrain perturbations, including visible overground obstacles, treadmill obstacles, visible drops, visible and invisible potholes, all suggest that leg angular trajectory is: (1) relatively insensitive to perturbations and (2) adjusted subtly over longer time-scales. This suggests a context-dependent feedforward optimization of leg angular trajectory at higher levels in the control hierarchy to enable robust and stable locomotion with minimal control intervention (Birn-Jeffery and Daley 2012; Birn-Jeffery et al. 2014).

Whereas leg angular trajectory appears insensitive to perturbations and adjusted over longer timescales, leg-length actuation shows high stride-to-stride variance, suggesting both predictive (feedforward) and reactive (feedback) adjustment in uneven terrain (Fig. 3; Birn-Jeffery and Daley 2012; Birn-Jeffery et al. 2014; Blum et al. 2014). Leg length actuation is sensitive to altered landing conditions, such that stance push-off is rapidly adjusted to stabilize the total mechanical energy of the body in uneven terrain. (Daley and Biewener 2006; Birn-Jeffery and Daley 2012; Birn-Jeffery et al. 2014; Blum et al. 2014). These findings suggest modular control of leg angular trajectory and leg-length actuation.

While modular control of leg angular trajectory and leg-length actuation have emerged as consistent control strategies for robustly stable running, it remains less clear whether, and under what circumstances, leg stiffness serves as a direct target of control. Research on humans running over soft and hard surfaces suggests that humans regulate leg stiffness to maintain steady body trajectory (Ferris et al. 1999). However, the specific terrain conditions used, soft and hard surfaces, did not allow a clear distinction between control priority for steady body trajectory versus consistent leg forces, because both were maintained. Humans running over visible downward steps exhibit anticipatory shifts in leg stiffness before a perturbation, but do not adjust leg stiffness within perturbed steps (Müller et al. 2012). In these human studies, subjects were specifically instructed to maintain constant running speed. In contrast, birds negotiating terrain drops exhibit high variance in leg stiffness while allowing speed to transiently vary (Daley et al. 2007; Blum et al. 2011; Müller et al. 2016). Whether or not leg stiffness is directly regulated may depend on the context-dependent constraints on the locomotor task.

Differences between birds and humans in stiffness regulation could also relate to leg morphology. Birds have a more crouched leg posture with four segments, in contrast to the vertically oriented three-segment leg configuration of humans. The limb morphology of birds may allow more flexible adjustment of leg posture to accommodate terrain variation, minimizing the need for active regulation of leg stiffness. This idea is supported by evidence from a study that directly compared control strategies in humans and birds from a model-based perspective (Blum et al. 2011). Birds exhibited a wider range of stable control solutions without adjusting leg stiffness, whereas humans are required to adjust leg stiffness to remain in the stable solution space (Blum et al. 2011). Additionally, this study showed that birds exhibited higher robustness to terrain height variation than humans, consistent with the more crouched posture enabling postural adjustments to minimize disturbances.

## *In vivo* muscle recordings reveal neuromuscular mechanisms underlying robust, stable, and agile locomotion

While external measures of body and limb mechanics can help reveal task-level locomotor control strategies, these measures do not reveal the underlying neuromuscular mechanisms. *In vivo* recordings of muscle force, length, and activation dynamics during perturbed locomotion can help reveal the relative contributions of intrinsic mechanical, feedback, and feedforward control mechanisms. These studies also help reveal how neuromechanics of locomotion are integrated across levels of organization, from individual muscle–tendon dynamics to joint, whole limb, and body dynamics. The relationship between muscle activation and mechanical output is known to be nonlinear and dynamically variable, depending on instantaneous fascicle length, velocity and recent strain history (Askew and Marsh 1998; Josephson 1999; Edman 2012; Herzog 2014). *In vivo* measures of muscle function during steady-state locomotor behaviors have revealed muscle–tendon mechanisms for economic bipedal locomotion (Biewener and Baudinette 1995; Roberts et al. 1997; Daley and Biewener 2003), but do not reveal the mechanisms underlying robustness, stability, and agility in non-steady behaviors.

**Fig. 4.**
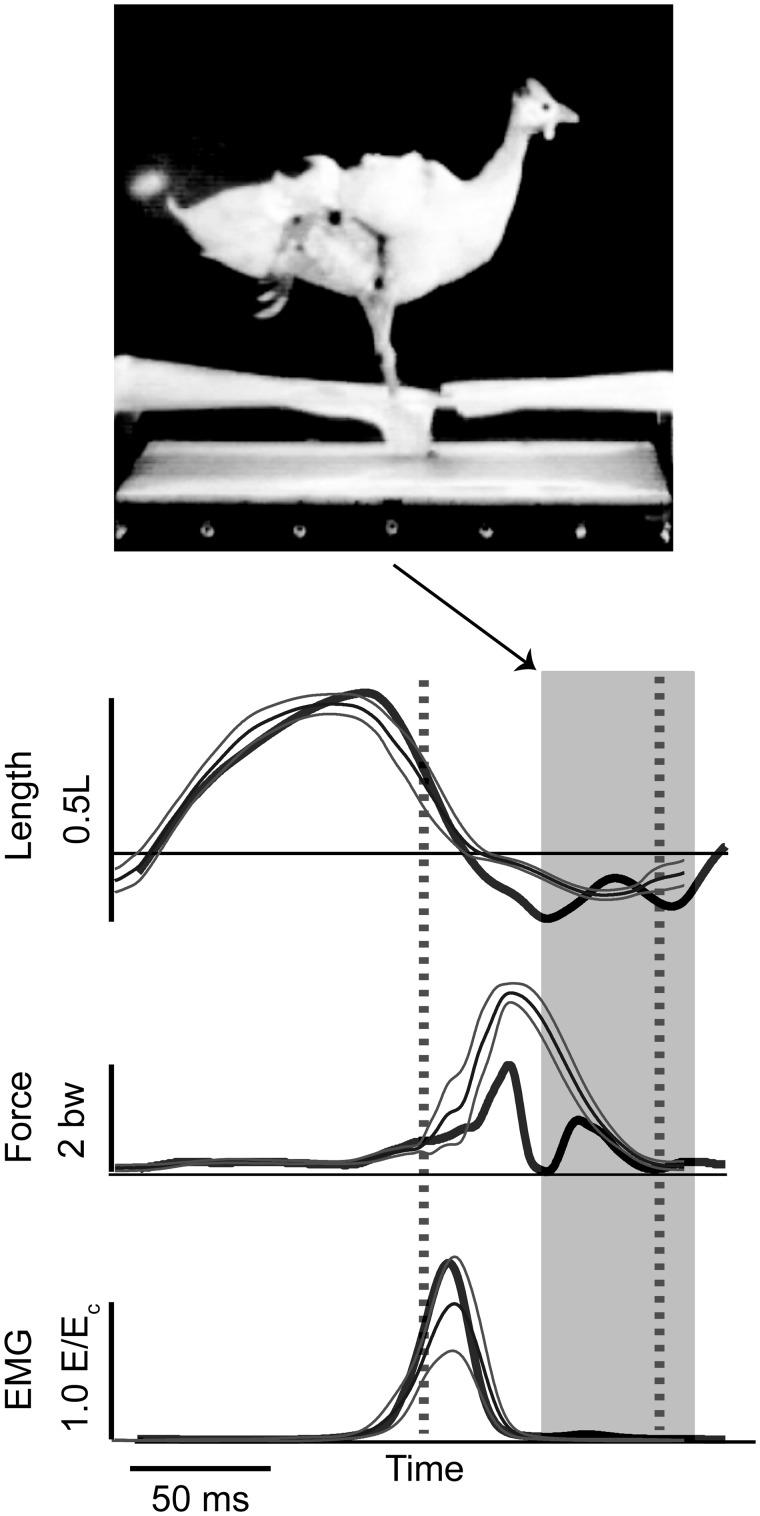
LG muscle length, force, and activation during the immediate response to a hidden pothole perturbation. Figure modified from Daley et al. (2009). At top, the guinea fowl is pictured at the time of ground contact after breaking through the false-floor of tissue paper. Below, thin lines indicate the mean and 95% confidence intervals for steady level running, and thick lines illustrate a perturbed drop step. Force and length are rapidly altered in response to the perturbation, although muscle activation (EMG) remains similar to the level terrain condition.

**Fig. 5.**
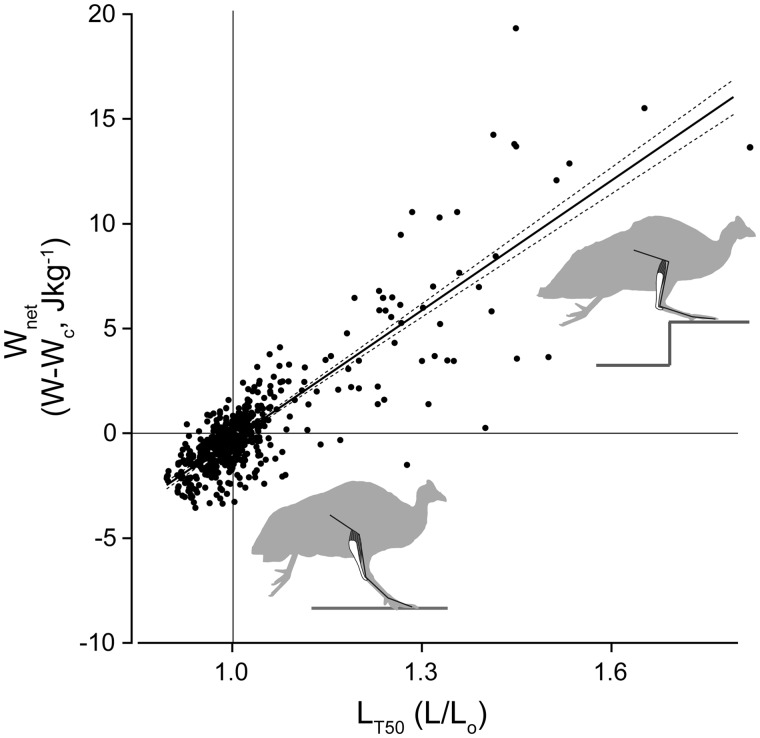
Load- and posture-dependent actuation of the LG muscle during negotiation of uneven terrain. When leg posture is altered at the time of foot contact, altering the balance between muscle and external forces, muscle length during force development (L_t50_) varies. L_t50_ is the largest predictor of the force and total work output of the muscle (W_net_) ( Daley et al. 2009, Daley and Biewener 2011 ). This posture-dependent response is similar between unexpected perturbations and repeating obstacles. This suggests similar task-level control strategies across context, despite potential for differing contributions of intrinsic mechanical, feedforward, and feedback control mechanisms to the response.

*In vivo* recordings of distal hindlimb muscles of the guinea fowl during negotiation of uneven terrain has shown that these muscles exhibit rapid changes in force and work in response to altered foot–substrate interactions, contributing to the intrinsic stability of locomotion. During negotiation of unexpected potholes, the peak force of the lateral gastrocnemius muscle (LG) during stance decreases by 81% during perturbed steps compared to steady strides, despite maintaining the same electromyography (EMG) activation levels (Fig. 4; Daley et al. 2009). The muscle shortens rapidly during the initial perturbation period, when the foot contacts and breaks through the false floor (tissue paper) and extends toward the true ground below (Fig. 4). In the subsequent stance period, peak muscle force is reduced, but peak ground reaction force is similar, and the muscle is stretched, resulting in energy absorption (Daley and Biewener 2006; Daley et al. 2009). This has a stabilizing effect on the body mechanical energy, offsetting the increase in kinetic energy gained through exchange of gravitational potential energy during the fall (Daley and Biewener 2006; Daley et al. 2009). A similar but converse response is observed in upward steps and obstacles, in which increased stretch and longer length during force development during a step onto an obstacle results in higher force production and work output, increasing mechanical energy of the body (Daley and Biewener 2011; Fig. 5). These studies revealed that LG force–length dynamics rapidly adjust the degree of stance push-off in response to altered foot–substrate interactions. This load-dependent actuation response of distal hindlimb muscles provides rapid stabilization of body mechanical energy in uneven terrain, and is consistent with observed whole-body and leg dynamics. Subsequent modelling studies have also confirmed that load-dependent actuation increases robustness and stability of running dynamics (Schmitt and Clark 2009).

Load- and posture-dependent shifts in muscle force and work occur without shifts in total muscle EMG activity during unexpected drop perturbations, revealing that intrinsic mechanisms play an important role in the response (Daley et al. 2009). However, increased EMG activity does contribute to the response during obstacle steps, likely mediated through short-latency proprioceptive reflexes (Daley and Biewener 2011). Interestingly, the qualitative patterns of muscle force–length dynamics remain similar in both unexpected and anticipated obstacle conditions (Daley et al. 2009; Daley and Biewener 2011). Nonetheless, while the overall force–length dynamics of the muscles remain similar across contexts, there is clear evidence of shifts in the relative contribution of intrinsic and neurally-mediated mechanisms of control, depending on the sensory context.

**Fig. 6.**
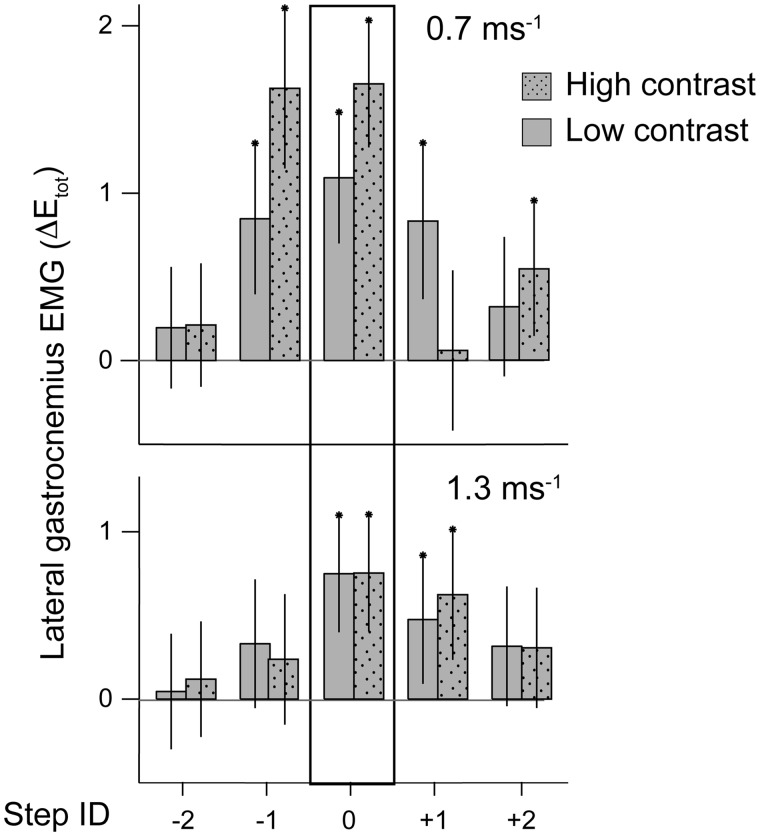
Context dependent-shifts in the contribution of predictive and reactive modulation of LG activity during obstacle negotiation. Guinea fowl running over obstacles on a treadmill encountered a single footfall on an obstacle (black box) approximately once in 5–7 steps. Step ID corresponds to the sequence of steps with the obstacle encounter at step zero. LG exhibits predictive increases in muscle activity at slower walking speeds (0.7 m/s). Predictive shifts are larger when the obstacles are more visible (higher contrast) relative to the level terrain. At higher speeds (1.3 m/s) guinea fowl use a reactive strategy, with increases in LG activity occurring after foot contact with the obstacle. The influence of high versus low contrast terrain is greater at slower speeds, when the bird has a longer time to process visual information to modulate muscle activity.

In a more recent study, Gordon and colleagues (2015) investigated context dependent shifts in sensorimotor control by comparing muscle activation patterns during obstacle negotiation at low and high speeds, and with low and high-contrast obstacles. In slower speed obstacle negotiation, anticipatory increases in muscle activity are apparent in the steps preceding obstacles. At higher running speeds, the neuromuscular response is largely reactive, occurring after foot contact with the obstacle (Fig. 6; Gordon et al. 2015). Anticipatory increases in muscle activity are larger when the obstacles are more easily visible (higher contrast to surrounding terrain), but mainly in slower speed obstacle negotiation. In the higher speed condition, the response remains mainly reactive, despite increased obstacle visibility (Gordon et al. 2015). This likely relates to the sensorimotor delays involved in visual contributions to path planning and navigation in higher brain centers. The results are consistent with a shift in sensorimotor control mechanisms with speed, with greater reliance on vision and anticipatory adjustments at slower speeds, and greater reliance on intrinsic mechanics and reactive feedback mechanisms at high speeds. Thus, the regulation of muscle dynamics reflects a redundant system with coordinated contributions from intrinsic mechanical, feedback, and feedforward mechanisms.

## Conclusions

While neuromechanical control of locomotion involves a complex interplay of mechanical and sensorimotor mechanisms, studies of running birds have revealed several strategies for robust, stable, and agile bipedal locomotion that are consistent across terrain contexts: (1) independent control of leg angular cycling and leg length actuation, which facilitates dynamic stability through simple control mechanisms, (2) feedforward regulation of leg cycling rate to maintain consistent leg loading in uneven terrain, (3) load-dependent muscle actuation to stabilize body mechanical energy in response to disturbances, and (4) multi-step recovery strategies that allow body dynamics to transiently vary while tightly regulating leg loading to minimize risks of fall and injury. Muscle proprioceptive feedback arising from non-steady force–length dynamics likely plays important roles in effective tuning of perturbation responses over time, as well as maintaining accurate state estimates for internal models, path planning, and navigation in higher brain centers. However, it remains unclear how sensory feedback is integrated with spinal neural circuits and higher brain centers to adjust locomotor control over short and long time-scales. In future work, it will be interesting to investigate the learning and adaptation of neuromechanical control mechanisms through repeated exposure to perturbations in controlled conditions.
